# Supporting Behavior Change in Sedentary Adults via Real-time Multidimensional Physical Activity Feedback: Mixed Methods Randomized Controlled Trial

**DOI:** 10.2196/26525

**Published:** 2022-03-02

**Authors:** Max James Western, Martyn Standage, Oliver James Peacock, Tom Nightingale, Dylan Thompson

**Affiliations:** 1 Department for Health University of Bath Bath United Kingdom; 2 School of Sport, Exercise and Rehabilitation Sciences University of Birmingham Birmingham United Kingdom

**Keywords:** physical activity, feedback, wearables, behavior change, sedentary time

## Abstract

**Background:**

Increasing physical activity (PA) behavior remains a public health priority, and wearable technology is increasingly being used to support behavior change efforts. Using wearables to capture and provide comprehensive, visually persuasive, multidimensional feedback with real-time support may be a promising way of increasing PA in inactive individuals.

**Objective:**

This study aims to explore whether a 6-week self-monitoring intervention using composite web-based multidimensional PA feedback with real-time daily feedback supports increased PA in adults.

**Methods:**

A 6-week, mixed methods, 2-armed exploratory randomized controlled trial with 6-week follow-up was used, whereby low to moderately active (PA level [PAL] <2.0) adults (mean age 51.3 years, SD 8.4 years; women 28/51, 55%) were randomly assigned to receive the self-monitoring intervention (36/51, 71%) or waiting list control (15/51, 29%). Assessment of PA across multiple health-harnessing PA dimensions (eg, PAL, weekly moderate to vigorous intensity PA, sedentary time, and steps), psychosocial cognitions (eg, behavioral regulation, barrier self-efficacy, and habit strength), and health were made at the prerandomization baseline at 6 and 12 weeks. An exploratory analysis of the mean difference and CIs was conducted using the analysis of covariance model. After the 12-week assessment, intervention participants were interviewed to explore their views on the program.

**Results:**

There were no notable differences in any PA outcome immediately after the intervention; however, at 12 weeks, moderate-to-large effects were observed with a mean difference in PAL of 0.09 (95% CI 0.02-0.15; effect size [Hedges *g*] 0.8), daily moderate-intensity PA of 24 (95% CI 0-45; Hedges *g*=0.6) minutes, weekly moderate-to-vigorous intensity PA of 195 (95% CI 58-331; Hedges *g*=0.8) minutes, and steps of 1545 (95% CI 581-2553; Hedges *g*=0.7). Descriptive analyses suggested that the differences in PA at 12 weeks were more pronounced in women and participants with lower baseline PA levels. Immediately after the intervention, there were favorable differences in autonomous motivation, controlled motivation, perceived competence for PA, and barrier self-efficacy, with the latter sustained at follow-up. Qualitative data implied that the intervention was highly informative for participants and that the real-time feedback element was particularly useful in providing tangible, day-to-day behavioral support.

**Conclusions:**

Using wearable trackers to capture and present sophisticated multidimensional PA feedback combined with discrete real-time support may be a useful way of facilitating changes in behavior. Further investigation into the ways of optimizing the use of wearables in inactive participants and testing the efficacy of this approach via a robust study design is warranted.

**Trial Registration:**

ClinicalTrials.gov NCT02432924; https://clinicaltrials.gov/ct2/show/NCT02432924

## Introduction

### Background

The health benefits of leading a physically active life are well-established with higher volumes of physical activity (PA), reducing the risk of numerous chronic diseases, mood disorders, and premature mortality [1-3]. In contrast, physical inactivity and prolonged sedentary time have been shown to be independent risk factors for noncommunicable conditions, including type 2 diabetes, cardiovascular disease, cancer, and musculoskeletal disease [4-6]. In addition to health and well-being ramifications, it is estimated that physical inactivity costs US $53.8 billion for health care systems around the world [7]. Collectively, such data stress the need for wide-reaching, cost-effective solutions. The availability, accuracy, and popularity of wearable technology for capturing PA behavior has surged in recent years and presents a potentially useful, affordable, and accessible tool for driving increases in PA levels [8,9]. However, commercial activity monitors are typically marketed at, and used by, young adults who have relatively high baseline PA levels as a means of monitoring exercise performance. Thus, the effectiveness of PA monitoring in inactive populations remains understudied and undetermined [10]. Sophisticated monitoring technology enables personalized motivational and persuasive feedback for individuals who would benefit from an increase in PA [11].

There are multiple dimensions of PA behavior that can independently affect health and well-being [12,13]. Analysis of wearable-derived data shows that individuals can score high and low on any number of these health-harnessing dimensions such as sedentary time, moderate to vigorous PA (MVPA), and overall energy expenditure [14,15], which could present a challenge when providing feedback on the appropriateness of one’s behavior. However, this understanding could also be beneficial, as each dimension can be presented as a unique opportunity for behavior change and, in principle, help individuals find bespoke solutions across a person’s day, which can help them overcome personal barriers or anchor them to their particular health goals [16]. Moreover, recipients can use reliable multidimensional PA feedback to understand and mitigate against compensatory changes in one aspect of their behavior in response to an attempt to alter another (eg, replacing moderate habitual activity with sedentary time in response to a new exercise regime). Preliminary qualitative data suggest that presenting multiple health-harnessing dimensions to adults is an acceptable, comprehensible, and motivating means of communication that could be readily implemented to support behavior change [17].

The Multidimensional Individualized Physical Activity (MIPACT) trial [18] examined whether a 12-week self-monitoring intervention incorporating multidimensional feedback alongside brief trainer support led to increases in PA behavior among adults at risk of chronic disease. After 3 and 12 months, there was very little change in behavior using this approach despite excellent compliance and adherence [19]. In MIPACT, participants received personal feedback on their multidimensional PA profile and both time spent and energy expended at different PA intensities via the manual upload of data from the monitor to a web-based *app* for viewing their behavior retrospectively. Although this approach is educational and might raise awareness about past behavior [20,21], other persuasive behavioral techniques to support ongoing, acute regulation of behavior or habit formation may be important precursors of sustained change [22,23].

Interventions that have used continuous *real-time* PA feedback (eg, pedometers) have shown promise in supporting changes in PA behavior [24-27]. By extending such work, real-time feedback provided across multiple PA dimensions might compliment a more holistic composite of PA feedback to provide both a *bigger picture* as well as a time-segmented appreciation of PA within the context of people’s daily lives. In other words, providing informative data about their progress toward a discrete and achievable activity target in real time can allow people to make quick behavioral adjustments and work toward their overall weekly health goal. The key to such an endeavor is the use of wearable technologies to provide informational feedback and primes based on real-time assessments so as to best capitalize on within-activity motivation *quality* [28].

To best use technological advancements to improve health and well-being, the use of an appropriate motivational theory is a necessity [28]. Self-determination theory (SDT) is a broad and empirically based theory of motivation that provides insight into how to translate informational feedback [29]. At the heart of the SDT is the proposition that people have 3 universal and essential necessities for wellness, healthy functioning, development, and growth, namely the satisfaction of the psychological needs of autonomy, competence, and relatedness [28]. In PA and exercise settings, empirical research has supported the role of need satisfaction in supporting high-quality forms of motivation (ie, autonomous, wherein intrinsic enjoyment and value of the behavior or identified congruence with self-identity guide behavior), better experiences, and higher well-being [28]. Research has also shown autonomous motivation toward exercise to positively predict objectively assessed exercise bouts [30].

Within SDT, it is postulated that when social inputs such as those inherent within interpersonal interactions or embedded in informational, *real-time* feedback satisfy basic psychological needs for autonomy, competence, and relatedness, people are motivated to act for high-quality reasons and experience greater well-being and better experiential outcomes [31]. Applied to the current work, the use of sophisticated PA data visualizations with light touch trainer support, self-monitoring, and real-time feedback was designed to support autonomy (eg, via the provision of choice, exploring new activities or options, and use of meaningful rationales), competence (eg, through the promotion of self-monitoring and clear, constructive, and relevant feedback), and relatedness (eg, demonstrating interest in people and acknowledging and respecting their perspectives and feelings).

### Objective

The primary aim of the present work is to explore whether the provision of sophisticated visual feedback with additional real-time feedback across multiple dimensions of PA supports changes in PA behavior. The secondary aims are to examine whether any changes in behavior lead to meaningful changes in health status over 12 weeks or whether any psychological variables change in response to the intervention. A supplementary aim is to explore the thoughts and feelings of intervention participants to further understand and explain their engagement with and impact of the program.

## Methods

### Study Design

To explore the efficacy of using combined, multidimensional, composite, and real-time PA feedback on behavior change, a pilot 12-week, 2-armed randomized controlled trial (RCT) design with quantitative and qualitative evaluation was used. The study was registered at ClinicalTrials.gov (NCT02432924) and received ethical approval from the University of Bath’s research ethics approval committee for health (reference number: EP 14/15 10). Study outcomes were assessed on 3 occasions. The first 2 assessments were taken before and after a 6-week self-monitoring intervention (or usual behavior if control), with the third assessment following a further 6-week follow-up period in which participants were without feedback. Control participants were offered a 6-week feedback intervention after their third assessment, whereas the intervention group participants were invited to undertake a one-on-one, semistructured interview to provide rich insights into their experience of the intervention.

### Participants

Participants were men and women aged between 40 and 70 years who responded to advertisements through the external university webpages, Twitter, and local newspaper articles for people who did not feel they were currently very active. All participants who inquired were sent a participant information sheet and subsequently screened for eligibility via a telephone call. Volunteers were deemed ineligible if they were actively being treated for a chronic disease that might have impeded their ability to change their PA (coronary heart disease, chronic kidney disease [stages 3-5], diabetes mellitus, stroke, heart failure, and peripheral arterial disease) or if they had a PA level (PAL; total energy expenditure divided by resting metabolic rate) of <2.0, which has been categorized by the World Health Organization as representing a highly active lifestyle [32]. The exploratory nature of this study meant that no formal sample size calculation was undertaken.

### Intervention

#### Waiting List Control Arm

The waiting list control group was encouraged to conduct their usual behavior until they had had 2 further assessments in line with those of the intervention group (ie, 6 and 12 weeks after randomization). At the time of revealing their allocation, waiting list participants were informed that upon completion of the third assessment, they would be able to receive the 6-week self-monitoring intervention in full (without any further follow-up assessment) but to carry on as normal in the meantime.

#### Intervention Arm (6-Week Active Intervention and 6-Week Follow-up)

**Figure 1:**
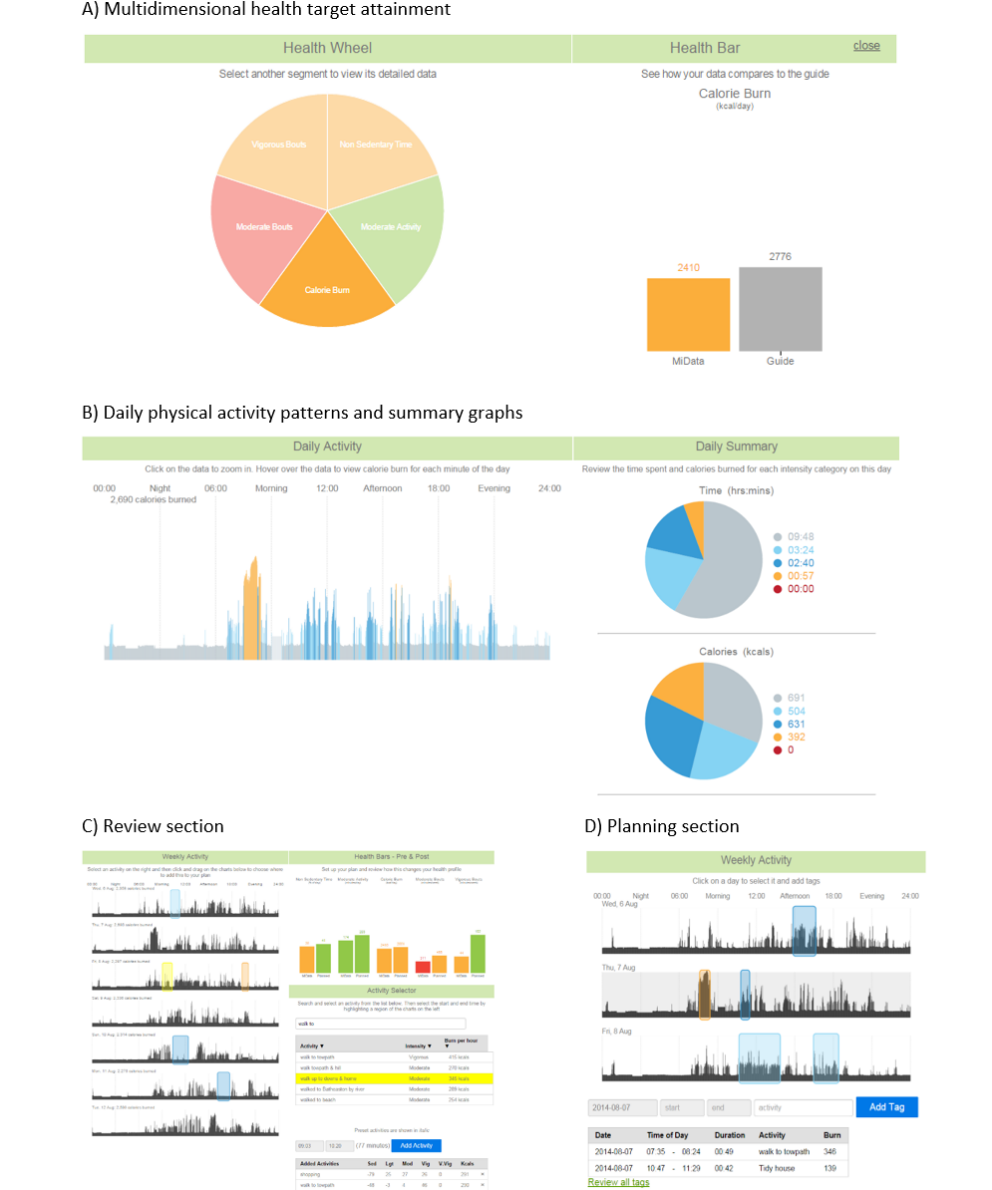
Features and examples of feedback and functions included on the Multidimensional Individualized Physical Activity web-based platform. (A) Participants were provided feedback using a traffic light–colored health target attainment schematic across the five dimensions and (B) detailed activity patterns and time use summaries colored in accordance with the intensity of activity during each given minute. (C) Participants were also able to review specific segments of a day to learn about the energy cost and intensity of particular activities and (D) were provided with a planning section where they could see how the addition of new activities, derived from the Ainsworth compendium [33], would affect their health targets if imposed over their existing week.

Participants randomized to the intervention group returned to the University of Bath at their earliest convenience to undertake a set-up session. Here, participants were shown multidimensional feedback on their weekly PA using the MIPACT web platform, as described by Peacock et al [18]. Briefly, the website provides informational feedback in the form of visual representations of their behavior across a 7-day period. To this end, the feedback encompasses five key health targets (Figure 1A): daily calorie burn, sedentary time, accumulated daily minutes of moderate-intensity activity, weekly MVPA in at least 10-minute bouts, and weekly vigorous-intensity activity accumulated in at least 10-minute bouts. Using a simplified and more detailed graphic, participants were shown each target attainment using a traffic light system where green would indicate a hit target, amber would indicate close to the target, and red would indicate a missed target.

Additional feedback was provided in the form of 24-hour PA patterns that were color coded to indicate the intensity of activity at a given minute of the day (Figure 1B). The web platform also included 2 interactive tabs whereby participants could tag activities to learn about and explore the specific intensity and energy expenditure of a given activity or period (Figure 1C) and forward plan future activities that could be superimposed on a given week’s PA patterns to visualize and explore the impact of adding new or existing activities on their health targets (Figure 1D). The Ainsworth Compendium of Physical Activities [33] was used as the basis for calculating the intensity category and personalized energy expenditure for each added activity in the menu of PAs.

For the real-time feedback element, participants were provided with a Bodymedia Mini (Sensewear; version 8.0) monitor, a smaller model that uses the same algorithms and sensors as the Bodymedia Core used for the assessments, and an accompanying real-time analog display that synced data directly from the armband. The small clip-on display provided feedback on daily accumulated minutes of moderate-intensity activity and minutes of vigorous-intensity activity, calories, and steps that were contextualized alongside the web platform’s moderate, vigorous, calorie burn, and nonsedentary time goals, respectively. In addition to real-time data, the display also stores the total 24-hour values for the previous day and enables users to set personalized targets for each of the 4 activity metrics. If targets were met, a congratulatory message was displayed on the screen, and an alarm sounded to inform the user of their success.

Participants were given an operating manual for the device and encouraged to use it as often as they felt necessary during the 6-week period. Over the course of the intervention period, the participant and researcher met a further 3 times to upload new data from the armband to the MIPACT web platform at weeks 2, 4, and 6. These 15-minute informal sessions afforded each participant the opportunity to troubleshoot any technical queries, get help interpreting their personal multidimensional web feedback, and discuss new plans of action for change. Each session was delivered in a need-supportive manner, encompassing the provision of participant choice; exploration of new activities or plans; and promotion of self-monitoring with clear, constructive, and relevant feedback while taking a clear interest in the perspectives and feelings of the participant [34].

### Measurement Procedures

#### Overview

The baseline laboratory session lasted approximately 45 minutes and involved the signing of informed consent, completion of a questionnaire pack, measurement of brachial seated blood pressure, measurement of anthropometric elements, and retrieval of a fasting venous blood sample from the antecubital vein. Participants were asked to attend the session having abstained from food or caffeine for a minimum of 10 hours. At the end of the session, participants were provided with a PA monitor and instructed to wear the device for 7 consecutive days, removing solely for water-based activities. Participants were also provided with a preaddressed envelope with which to return the activity monitor. The Index of Multiple Deprivation was calculated using participants’ postcode on the UK government English indices of deprivation webtool [35], and deprivation decile was extracted for each participant [36]. All procedures other than the signing of informed consent were replicated at the 6- and 12-week follow-up assessment time points.

#### Primary Outcome: PA

PA was measured using the Bodymedia Mini (Sensewear; version 8.0), which has been shown to accurately measure minute-by-minute energy expenditure [37,38]. To be included in the analysis, participants required a minimum of 6 valid days that included 80% of an assumed 16-hour waking day. On occasions where participants removed the device during sleep or at other times, the estimated resting metabolic rate [39] was assigned to missing data points to complete the 24-hour period. Minute-by-minute energy expenditure was used to determine time (minutes) spent in each of the activity intensity thresholds (sedentary: <1.8 metabolic equivalent of tasks [METs]; light: ≥1.8 and <3.0 METs; moderate: ≥3.0 and <6.0 METs; vigorous: ≥6.0 METs) [40]. These data were used to determine changes in each of the key health-harnessing PA dimensions used in the feedback, including (1) PAL (total energy expenditure divided by resting metabolic rate), (2) sedentary time (percentage of waking day) and accumulated 1-minute bouts of moderate-intensity activity (minutes per day), (3) MVPA accumulated in bouts of ≥10 minutes (per week), and (5) vigorous-intensity activity accumulated in bouts of ≥10 minutes (per week). The mean daily steps were also determined for each assessment.

#### Secondary Outcomes: Health Markers

Blood pressure was measured using an automatic sphygmomanometer immediately after 15 minutes of isolated rest. A total of 3 measurements were taken at least 1 minute apart, and the mean of the readings was used as the recorded value. Height was measured without shoes to the nearest millimeter using a Seca stadiometer and weight to the nearest 100 g using a set of digital Tanita (BC-543) scales. These measures were used to calculate BMI (kg/m^2^) for each participant. Waist circumference measurements were taken to the nearest millimeter using a Hoechstmass tape measure placed parallel to the floor at the midpoint between the iliac crest and the lowest palpable rib after gentle exhalation. The mean of 3 measurements was taken provided they were within 0.5 cm of one another. A 10 mL fasted venous blood sample was taken at each assessment and used to measure concentrations of plasma glucose, insulin, total cholesterol, high-density lipoprotein cholesterol, low-density lipoprotein cholesterol, triglycerides, and C-reactive protein. These metabolic biomarkers were quantified using commercially available spectrophotometric assays (Randox Laboratories, Co) and enzyme-linked immunosorbent assay (serum insulin only: Mercodia AB). The homeostasis model assessment calculator was used to estimate insulin resistance (Homeostatic Model Assessment of Insulin Resistance-2).

Each participant also completed the EuroQol (EQ) 5-dimension 5-level questionnaire [41], which measures the quality of life across five dimensions: mobility, self-care, usual activities, pain or discomfort, and anxiety or depression. The EQ visual analog scale was used to record patients’ overall perception of their health from 0 (worst imaginable) to 100 (best imaginable). The Short Form-36 (SF-36) Health Survey Questionnaire was used to determine any changes in perceived physical and mental health [42]. In total, eight health concepts were measured by the SF-36, with four scales each loaded onto two higher-order factors: physical (physical functioning, physical impact on role, bodily pain, and general health) and mental (ie, vitality, social functioning, emotional impact on role, and mental health) health [43]. Using the standardized scoring algorithms outlined by Ware et al [43], component summary scores were computed for physical and mental health ranging from 0 to 100, with higher scores representing better health status.

#### Secondary Outcomes: Motivation and Psychological Variables

The questionnaire pack included a collection of instruments for which the reliability and validity of the scores have been described at length by the respective cited authors. Where necessary, the stem of the respective questions was altered from its original wording to refer to *PA* rather than *exercise*. To measure participants’ motivation, as propagated within SDT, the Psychological Need Satisfaction in Exercise scale [44] was used to measure autonomy, competence, and relatedness, and the Behavioral Regulation in Exercise Questionnaire-2 [45] was used to explore the participants’ motivation to engage in PA (ie, autonomous and controlled reasons). Perceived competence in PA [46] was also included as a more specific measure of an individual’s self-belief. The Barrier Self-Efficacy scale [47] was included to determine whether the intervention changed people’s confidence to undergo PA in the face of common obstacles, and the Self-Report Habit Index [48] was used to determine the automaticity of PA behavior. The Subjective Vitality Scale [49] was used to detect changes in vitality.

#### Postintervention Interviews

Participants who successfully completed the intervention were invited to attend a one-on-one semistructured interview to discuss their experience with the program once all follow-up assessments were completed. The topic guide for these interviews (shown in full in Multimedia Appendix 1) included questions to capture participants’ views on the utility and retrospective and prospective impact of the intervention for them and unpick the aspects that were most useful and those that might be improved. The interviews typically lasted between 15 and 25 minutes and were recorded using an Olympus digital voice recorder. In addition, all intervention participants completed a feedback form that included rating scales for aspects of the real-time display (overall, personal targets, calories, steps, moderate and vigorous activity) and web-based feedback (overall, health targets, activity patterns, review function, and planning function). Scores ranged from 1=not useful at all and 3=somewhat useful to 5=extremely useful, with a 0 option if the element in question was not used.

### Analysis

Mean differences between intervention and control group participants for 6- and 12-week PA and 95% CIs across each of the 6 feedback dimensions were calculated using an analysis of covariance model [50]. Covariates included baseline values of each outcome variable to control for chance imbalances at baseline (accounting for any unequal variance because of unequal group allocation) and the factors used in balancing the groups (sex and weight status) [51]. Bias-corrected and accelerated bootstrapping was used to verify CIs via 5000 replications, as this approach has been recommended to provide more accurate estimates of SEs and CIs with smaller sample sizes [52-54]. The same analysis was used to explore differences in health outcomes and psychosocial variables at 6 and 12 weeks. Effect sizes (Hedges *g*) are provided for the mean difference between intervention and control across each variable and are interpreted as 0.2, 0.5, and 0.8 for small, moderate, and large effects, respectively [55]. A post hoc subgroup analysis to explore interactions with covariates observed at 12 weeks was performed, whereby unadjusted means and SDs were calculated to explore whether male versus female and participants with low versus high baseline PA had more pronounced changes in PA data.

Qualitative interviews were interpreted using descriptive deductive and inductive qualitative analyses based on the principles of thematic analysis [56]. Audio files were transcribed verbatim and uploaded to NVivo (version 11; QRS International) for coding and analysis. The lead author, who conducted the interviews, reread through each participant transcript for familiarization and then coded themes within the data. When all transcripts were coded, the themes were compared among participants, and common recurring viewpoints and other important insights were presented in the *Results* section as themes.

## Results

### Participants

**Figure 2:**
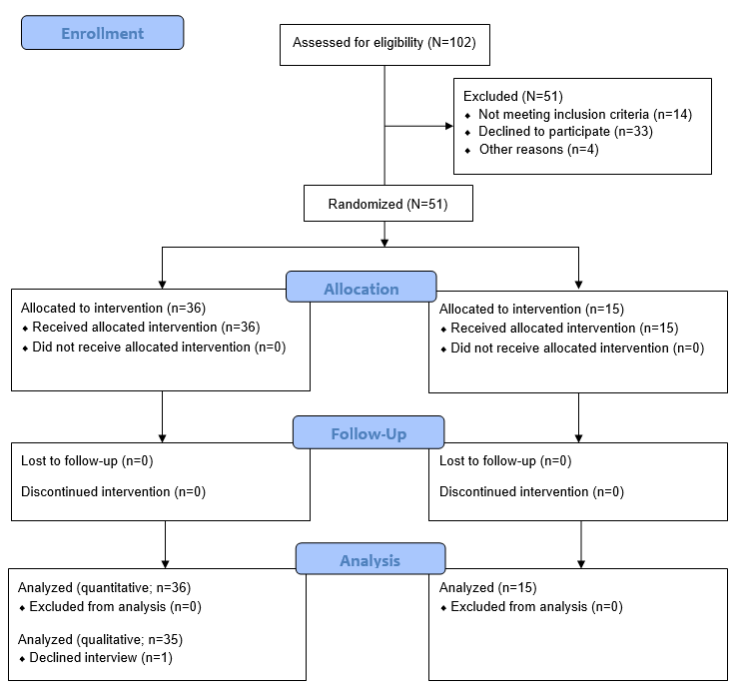
CONSORT (Consolidated Standards of Reporting Trials) flow diagram demonstrating participants’ progress through the study.

Figure 2 shows the flow of the participants through the study. Of the 102 inquiries, 57 (55.9%) participants were eligible, of whom 5 (9%) were excluded for being too active (PAL ≥2.0) at baseline, and 1 (2%) withdrew because of an allergic reaction to the PA assessment device; therefore, 51 (89%) participants were randomized into either the intervention group or the waiting list control group in a 2:1 allocation ratio to learn more about the intervention. A statistician external to the research team completed randomization and did not disclose any of the details before the completion of recruitment. The statistician stratified the participants by sex (male or female) and weight status (with BMI ≥30 kg/m^2^ as the binary cutoff point) using a block size of 6 (which was revealed to the researcher team after the study), giving an overall allocation of 36:15 in favor of the intervention group. No participant withdrew from the study, although one of the intervention participants declined to undergo the end-of-intervention interview. The baseline characteristics of the participants are displayed in Table 1.

**Table 1:** Baseline characteristics of study participants (N=51).

Characteristic		All	Intervention (n=36)	Control (n=15)
Age (years), mean (SD)		51.3 (8.4)	52.3 (8.2)	50.1 (8.3)
**Age (years), n (%)**				
	40-55	33 (65)	23 (64)	10 (67)
	55-70	18 (35)	13 (36)	5 (33)
Female		28 (55)	20 (55)	8 (53)
Ethnicity (White British)		46 (90)	32 (88)	14 (93)
**Marital status, n (%)**				
	Married or cohabiting	42 (82)	30 (83)	12 (80)
	Single, divorced, or widowed	9 (18)	6 (17)	3 (20)
**Education, n (%)**				
	GCSE^a^	3 (6)	3 (8)	0 (0)
	A-level	4 (8)	3 (8)	1 (7)
	First degree	24 (47)	17 (47)	7 (47)
	Higher degree	20 (39)	13 (36)	7 (47)
Index of Multiple Deprivation^b^ (decile), mean (SD)		8.0 (2.4)	7.9 (2.3)	8.2 (2.6)
**Index of Multiple Deprivation (decile), n (%)**				
	1-5	11 (22)	8 (23)	3 (20)
	6-10	39 (78)	27 (77)	12 (80)
Smoker		2 (4)	0 (0)	2 (13)

^a^GCSE: General Certificate of Secondary Education.

^b^Index of Multiple Deprivation based on postcode calculated [35].

### Primary Outcome: PA

All 51 participants provided complete PA data at the 6- and 12-week time points and baseline and were therefore included in the exploratory analysis of the primary outcome. The baseline characteristics of the participants are shown in Table 1. The total 24-hour wear time across the week for the 3 assessment time points was, on average, 98% (SD 1.6%), 96% (SD 8.2%), and 95% (SD 8.1%) in the intervention group and 95% (SD 7.9%), 95% (SD 8.7%), and 94% (SD 12.4%) in the control group, respectively. Table 2 shows the adjusted mean difference (95% CIs) between the intervention and control groups at the 6- and 12-week time points and effect sizes for each PA outcome.

There were no observed differences in any PA outcomes at the 6-week end-of-intervention assessment. At 12 weeks, relative to control participants, the intervention group had reduced mean daily sedentary time by −40 (95% CI −76 to −4) min/day and increased light-intensity activity by 14 (95% CI −78 to 45) minutes per day, moderate-intensity activity by 22 (95% CI 1-45) minutes per day, and vigorous-intensity activity by 2 (95% CI −1 to 6) minutes per day. Post hoc descriptive analysis of subgroups indicated that changes in PA were more pronounced in female participants than in males and for individuals with lower baseline PA levels at 12 weeks (Multimedia Appendix 2, Tables S1 and S2).

**Table 2:** Mean scores, adjusted mean difference between intervention and control groups, and effect sizes (with 95% CIs) across physical activity dimensions at 6 and 12 weeks.

Outcome and time point			Intervention (n=36)^a^, mean (95% CI)		Control (n=15)^a^, mean (95% CI)		Adjusted mean difference^a,b^ (95% CI)		Effect size, Hedges *g* (95% CI)
**PAL^c,d^ (TEE^e^ divided by RMR^f^)**									
	Baseline	1.61 (1.55 to 1.66)		1.62 (1.55 to 1.68)		N/A^g^		N/A	
	Week 6	1.62 (1.57 to 1.67)		1.65 (1.58 to 1.72)		−0.02 (−0.10 to 0.04)		−0.2 (−0.8 to 0.4)	
	Week 12	1.67 (1.63 to 1.72)		1.58 (1.52 to 1.64)		0.09 (0.02 to 0.15)		0.8 (0.2 to 1.4)	
**Sedentary time^h^ (percentage waking day)**									
	Baseline	69 (66 to 73)		69 (64 to 73)		N/A		N/A	
	Week 6	69 (66 to 72)		66 (62 to 70)		3 (−2 to 8)		0.3 (−3 to 0.9)	
	Week 12	66 (63 to 69)		70 (65 to 74)		−4 (−8 to 1)		−0.5 (–1.1 to 0.1)	
**Moderate activity^i^ (minutes per day)**									
	Baseline	111 (94 to 129)		117 (99 to 135)		N/A		N/A	
	Week 6	118 (105 to 130)		127 (107 to 148)		−10 (−28 to 8)		−0.3 (−0.9 to 0.3)	
	Week 12	132 (118 to 147)		109 (89 to 131)		24 (0 to 45)		0.6 (0.0 to 1.2)	
**Vigorous bouts^j^ (minutes per week)**									
	Baseline	42 (23 to 65)		26 (12 to 43)		N/A		N/A	
	Week 6	48 (30 to 70)		46 (24 to 71)		2 (−24 to 28)		0.0 (−0.6 to 0.6)	
	Week 12	50 (30 to 73)		33 (14 to 55)		18 (−5 to 41)		0.4 (−0.2 to 1.0)	
**MVPA^k^ bouts^j^ (minutes per week)**									
	Baseline	539 (435 to 646)		509 (400 to 622)		N/A		N/A	
	Week 6	584 (495 to 675)		580 (441 to 725)		4 (−126 to 136)		0.0 (−0.6 to 0.6)	
	Week 12	658 (571 to 750)		462 (340 to 587)		195 (58 to 331)		0.8 (0.2 to 1.4)	
**Steps^l^ (steps per day)**									
	Baseline	7403 (6705 to 8093)		7767 (6626 to 8884)		N/A		N/A	
	Week 6	8207 (7269 to 9114)		8280 (7268 to 9114)		−73 (−1122 to 1017)		0.0 (−0.6 to 0.6)	
	Week 12	8782 (7987 to 9656)		7236 (6496 to 7991)		1545 (581 to 2553)		0.7 (0.1 to 1.3)	

^a^CIs were verified using a bias-corrected and accelerated bootstrap with 5000 replications.

^b^Covariates included stratified randomization factors (BMI at baseline and sex) and baseline scores for the respective outcome variables.

^c^PAL: physical activity level.

^d^Mean total daily energy expenditure divided by daily resting metabolic rate.

^e^TEE: total energy expenditure.

^f^RMR: resting metabolic rate.

^g^N/A: not applicable.

^h^Percentage of waking day.

^i^All minutes ≥3 metabolic equivalents of task.

^j^Activity ≥6 metabolic equivalents of task (vigorous) or ≥3 metabolic equivalents of task (MVPA) accumulated in ≥10 minutes was counted.

^k^MVPA: moderate to vigorous physical activity.

^l^Mean daily step count.

### Secondary Outcomes: Health and Well-being

There were no 6- or 12-week differences in any of the cardiometabolic health outcomes measured between the intervention and control groups, except for insulin resistance calculated at week 12. The mental health component summary of the SF-36 improved in the intervention group at 12 weeks; however, neither the SF-S6 nor the physical component summary, EQ 5-dimension 5-level questionnaire, or visual analog scale scores were different at any other time point. The baseline, 6-week, and 12-week scores for all variables are shown in Table 3.

**Table 3:** Secondary health and psychosocial outcomes at 6 and 12 weeks (N=51)^a^.

Outcome and week				Baseline, mean (SD)		Intervention, mean (95% CI^b^)		Control, mean (95% CI^b^)		Adjusted mean difference (95% CI^b^)		Effect size (Hedges *g*)
**Health and well-being**												
	**Systolic blood pressure (mm Hg)**											
		6 weeks	124 (14)		123 (118 to 128)		123 (118 to 128)		0.2 (–4.69 to 4.86)		0.02	
		12 weeks	124 (14)		124 (120 to 127)		120 (113 to 127)		4.33 (–3.33 to 11.43)		0.43	
	**Diastolic blood pressure (mm Hg)**											
		6 weeks	86 (10)		86 (83 to 90)		88 (83 to 93)		−1.53 (–6.03 to 3.21)		−0.20	
		12 weeks	86 (10)		88 (85 to 91)		86 (81 to 91)		1.85 (–3.88 to 7.79)		0.21	
	**Body mass (kg)**											
		6 weeks	81.9 (14.4)		81.6 (77.8 to 85.4)		82.3 (78.6 to 86.1)		−0.74 (–1.86 to 0.49)		−0.44	
		12 weeks	81.9 (14.4)		81.8 (78.2 to 85.4)		82.6 (79 to 86.4)		−0.81 (−2 to 0.36)		−0.33	
	**Waist circumference (cm)**											
		6 weeks	91.8 (11.9)		89.9 (86.6 to 93.3)		90.8 (87.3 to 94.4)		−0.93 (–2.47 to 0.81)		−0.32	
		12 weeks	91.8 (11.9)		89.2 (86.1 to 92.4)		89.2 (85.7 to 92.9)		0.01 (–1.67 to 1.77)		0.00	
	**Glucose (mmol/L)**											
		6 weeks	5.3 (0.7)		5.3 (5.1 to 5.5)		5.4 (5.2 to 5.6)		−0.08 (−0.32 to 0.17)		−0.22	
		12 weeks	5.3 (0.7)		5.3 (5.1,5.5)		5.5 (5.3 to 5.7)		−0.15 (−0.43 to 0.13)		−0.36	
	**Insulin (mIU/mL)**											
		6 weeks	6.7 (3.8)		6.4 (5.2 to 7.7)		6.6 (5.3 to 8)		−0.18 (–1.85 to 1.7)		−0.06	
		12 weeks	6.7 (3.8)		6.1 (5.1 to 7.3)		7.3 (6 to 8.9)		−1.25 (−2.4 to −0.16)		−0.50	
	**Insulin resistance**											
		6 weeks	1.6 (1.1)		1.5 (1.2 to 1.9)		1.6 (1.3 to 2.0)		–0.07 (−0.48 to 0.34)		−0.09	
		12 weeks	1.6 (1.1)		1.5 (1.2 to 1.7)		1.8 (1.5 to 2.1)		−0.34 (−0.61 to −0.62)		−1.23	
	**Total cholesterol (mmol/L)**											
		6 weeks	5.6 (0.8)		5.3 (5 to 5.6)		5.5 (5.1 to 5.9)		−0.16 (−0.56 to 0.22)		−0.23	
		12 weeks	5.6 (0.8)		5.4 (5.2 to 5.6)		5.5 (5.1 to 6)		−0.14 (−0.65 to 0.32)		−0.23	
	**HDL^c^ cholesterol (mmol/L)**											
		6 weeks	1.3 (0.4)		1.4 (1.2 to 1.5)		1.4 (1.3 to 1.5)		−0.04 (−0.17 to 0.11)		−0.18	
		12 weeks	1.3 (0.4)		1.4 (1.2 to 1.5)		1.4 (1.3 to 1.6)		−0.06 (−0.17 to 0.04)		−0.38	
	**LDL^d^ cholesterol (mmol/L)**											
		6 weeks	3.7 (0.8)		3.5 (3.2 to 3.7)		3.5 (3.2 to 3.8)		−0.07 (−0.38 to 0.25)		−0.11	
		12 weeks	3.7 (0.8)		3.5 (3.3 to 3.7)		3.5 (3.1 to 3.9)		0.01 (−0.39 to 0.36)		0.03	
	**Triglycerides (mmol/L)**											
		6 weeks	1.4 (0.8)		1.2 (1 to 1.4)		1.3 (1.1 to 1.6)		−0.12 (−0.39 to 0.14)		−0.27	
		12 weeks	1.4 (0.8)		1.2 (1.1 to 1.4)		1.5 (1.2 to 1.7)		−0.23 (−0.55 to 0.08)		−0.54	
	**CRP^e^ (mg/L)**											
		6 weeks	2.0 (2.6)		2.4 (1.5 to 3.5)		1.6 (0.9 to 2.2)		0.89 (1.11 to 0.18)		0.32	
		12 weeks	2.0 (2.6)		1.9 (1.3 to 2.7)		3 (1.6 to 4.5)		−1.09 (–2.8 to 0.51)		−0.48	
	**EQ-5D VAS^f^**											
		6 weeks	65.2 (16.3)		72.2 (66.8 to 77.3)		71.7 (63.4 to 79.6)		0.45 (–7.57 to 8.45)		0.03	
		12 weeks	65.2 (16.3)		69.5 (62.5 to 75.7)		71.7 (66.6 to 76.7)		−2.12 (–11.23 to 6.47)		−0.12	
	**EQ-5D-5L^g^ score**											
		6 weeks	0.90 (0.1)		0.92 (0.89 to 0.95)		0.89 (0.85 to 0.93)		0.03 (−0.01 to 0.07)		0.38	
		12 weeks	0.90 (0.1)		0.89 (0.85 to 0.92)		0.9 (0.85 to 0.95)		−0.01 (−0.06 to 0.04)		−0.16	
	**SF-36^h^ physical health**											
		6 weeks	47.4 (8.4)		51.1 (48.3 to 53.9)		48.5 (45.2 to 51.5)		2.52 (–1.63 to 7.17)		0.39	
		12 weeks	47.4 (8.4)		47.5 (43.8 to 51.1)		50.1 (46.3 to 53.5)		−2.6 (–7.58 to 2.79)		−0.28	
	**SF-36 mental health**											
		6 weeks	49.0 (9.8)		50.8 (48.4 to 53)		48.9 (45.3 to 52.6)		1.86 (–2.29 to 6.03)		0.26	
		12 weeks	49.0 (9.8)		51.7 (47 to 56.3)		43.8 (38 to 49.4)		7.93 (0.74 to 15.18)		0.60	
**Motivation and psychosocial**												
	**Autonomous motivation**											
		6 weeks	2.9 (0.7)		3.1 (3 to 3.3)		2.9 (2.7 to 3)		0.26 (0.04 to 0.49)		0.79	
		12 weeks	2.9 (0.7)		3.1 (2.9 to 3.3)		3 (2.8 to 3.2)		0.1 (−0.1 to 0.3)		0.30	
	**Controlled motivation**											
		6 weeks	1.5 (0.7)		1.4 (1.2 to 1.6)		1.7 (1.4 to 1.9)		−0.28 (−0.55 to −0.01)		−0.63	
		12 weeks	1.5 (0.7)		1.3 (1.2 to 1.5)		1.6 (1.3 to 1.8)		−0.2 (−0.5 to 0.09)		−0.42	
	**Overall need satisfaction**											
		6 weeks	4.7 (1.0)		4.6 (4.2 to 4.9)		4.6 (4.3 to 5)		−0.03 (−0.57 to 0.39)		−0.03	
		12 weeks	4.7 (1.0)		4.7 (4.5 to 4.9)		4.6 (4.2 to 4.9)		0.12 (−0.21 to 0.43)		0.16	
	**Autonomy**											
		6 weeks	5.4 (0.6)		5.3 (5.1 to 5.5)		5.4 (5.1 to 5.7)		−0.07 (−0.39 to 0.26)		−0.12	
		12 weeks	5.4 (0.6)		5.5 (5.3 to 5.7)		5.6 (5.4 to 5.7)		−0.09 (−0.32 to 0.16)		−0.17	
	**Competence**											
		6 weeks	4.1 (1.2)		4.5 (4.2 to 4.7)		4.1 (3.7 to 4.5)		0.36 (−0.1 to 0.77)		0.47	
		12 weeks	4.1 (1.2)		4.3 (3.9 to 4.6)		4 (3.5 to 4.4)		0.32 (−0.18 to 0.8)		0.36	
	**Relatedness**											
		6 weeks	4.3 (1.3)		4.2 (3.7 to 4.6)		4.5 (4.1 to 4.9)		−0.35 (−0.88 to 0.16)		−0.37	
		12 weeks	4.3 (1.3)		4.4 (3.9 to 4.8)		4.4 (3.9 to 4.8)		0 (−0.74 to 0.72)		0.00	
	**Barrier self-efficacy**											
		6 weeks	49.7 (16.5)		52.3 (47.6 to 57)		41 (35.1 to 46.8)		11.35 (3.24 to 19.37)		0.84	
		12 weeks	49.7 (16.5)		53.3 (48 to 58.8)		43.9 (37.3 to 50.3)		9.38 (1.67 to 17.18)		0.68	
	**Vitality**											
		6 weeks	4.4 (1.1)		5.1 (4.8 to 5.4)		4.3 (3.7 to 4.9)		0.77 (0.17 to 1.37)		0.81	
		12 weeks	4.4 (1.1)		5.1 (4.7 to 5.5)		4.5 (3.8 to 5.1)		0.57 (−0.04 to 1.2)		0.51	
	**Perceived competence**											
		6 weeks	5.0 (1.3)		5.3 (4.9 to 5.6)		4.8 (4.4 to 5.1)		0.51 (0.14 to 0.92)		0.70	
		12 weeks	5.0 (1.3)		5.2 (4.8 to 5.7)		5 (4.6 to 5.5)		0.2 (−0.41 to 0.9)		0.19	
	**Habit**											
		6 weeks	1.6 (1.0)		2.1 (1.8 to 2.4)		1.5 (1.2 to 1.9)		0.56 (0.16 to 0.97)		0.84	
		12 weeks	1.6 (1.0)		2.1 (1.8 to 2.3)		1.7 (1.3 to 2)		0.41 (−0.04 to 0.86)		0.58	

^a^Covariates include baseline score for each parameter (as indicated in the pooled mean baseline column), BMI, and sex.

^b^CIs verified using bias-corrected and accelerated bootstrapping with 5000 repetitions.

^c^HDL: high-density lipoprotein.

^d^LDL: low-density lipoprotein.

^e^CRP: C-reactive protein.

^f^EQ-5D VAS: EuroQol 5-dimension visual analog scale.

^g^EQ-5D-5L: EuroQol 5-dimension 5-level questionnaire.

^h^SF-36: Short Form-36.

### Secondary Outcomes: Motivation and Psychosocial Variables

Relative to the control group, the intervention group had a reduction in controlled behavioral regulation (external and introjected regulation), and increases in autonomous behavioral regulation (intrinsic, integrated and identified regulation), perceived competence for PA and habit strength at the 6-week assessment but not at 12 weeks. Barrier self-efficacy was increased in the intervention group at 6 weeks and was sustained at the 12-week follow-up. Subjective vitality was also increased in the intervention group at 6 weeks but was not sustained until 12 weeks. No changes in overall psychological need satisfaction or its subscales were observed (Table 3).

### Intervention Component Evaluation

Participants were asked to provide their subjective ratings of the *usefulness* of intervention features at the 6-week assessment and qualitative feedback following their 12-week assessment. From the subjective ratings participants ranked the real-time display (mean 4.5, SD 0.8) higher than the web-based MIPACT platform (mean 3.3, SD 1.5) using a scale from 1 *not useful at all* to 5 *very useful*, or 0=*not used*. Each aspect of the real-time display consistently rated as more useful than the features of the web platform. Specifically, the display of calorie data (mean 4.0, SD 0.9), steps (mean 4.3, SD 0.7), moderate-vigorous activity (mean 4.3, SD 1.2) and having personal targets (mean 4.0, SD 1.5) was rated higher than the composite health target (mean 3.6, SD 1.5) and activity pattern (mean 3.7, SD 1.5) data. The lowest-ranked features were the more interactive web-based tools, namely the review (mean 2.3, SD 2.0) and planning (mean 2.0, SD 1.9) sections of the MIPACT website.

### Qualitative Evaluation

Qualitative feedback offers further insight into these ratings. Textbox 1 provides a summary of the key themes identified in the analysis of intervention participants’ interviews and quotations to illustrate each theme. All intervention participants championed the feedback as useful for raising their consciousness and awareness of their own PA behavior, with many mentioning an improved understanding of the time they spent inactive (theme 1). More than half of the intervention participants postulated that PA was now more of a priority after having been through the program and that it reinforced their belief that PA was a means of improving health (theme 2). The self-monitoring element helped individuals gauge how much PA was required to meet certain health recommendations (theme 3). According to many of the participants, the program inspired them to increase their PA levels, and two-thirds alluded to the fact that the multidimensional nature of the feedback assisted them in finding personal solutions (theme 4). Some participants said that during the 6-week program, they would consciously go out of their way to achieve the targets, and many put added emphasis on steps as a key and achievable daily motivator (theme 5).

**Key themes identified in the qualitative analysis and example quotations (participant information provided as sex, age, baseline physical activity level).**

**Theme 1: personalized feedback improved understanding of one’s own behavior**

- *I think it’s, it’s changed, it’s changed my day-to-day activity, and I am a lot more conscious of the fact that I am sitting a lot, and part of it, there was a realisation that I wasn’t very active.* [male, 46 years, 1.48]
- *Yeah, I think I was probably overestimating what I was doing, I thought I was more active than I was in a way so...when you see it’s like oh you are actually doing as much as I thought I’d probably on my feet but I’m not necessarily so doing anything that is going to benefit me stop so yeah it’s definitely made me more aware of the need.* [female, 42 years, 1.42]

**Theme 2: physical activity is now more of a priority or reinforced importance**

- *Um, well it certainly hasn’t become any less important. I probably would say that it has become more important because the awareness breeds that sort of feeling, you know, that this is something that is not just a one-off, you know. Over a three-month period, it’s, it’s life and it should continue.* [male, 59 years, 1.72]
- *And then hopefully, my hope is, as ie, as I lose weight...Because that’s one thing I haven’t done is lost weight...um, is once I have lost more weight that I will feel fitter and then I can up that target. But I don’t want to try and do too much, too soon.* [female, 62 years, 1.25]

**Theme 3: feedback helped people understand how to meet recommendations**

- *I found it interesting, you know? Because I know how many steps it takes me to go down our town and round and back to the house it’s at about 1800, I think. And I know how many is to go to the railway and things like that.* [female, 63 years, 1.37]
- *But of course, that whole thing then tipped me nicely over and I was...So, it had that useful upturn, and equally, as I said before, it helps me gauge just exactly how much distance I need to be covering to meet a, sort of a standard target.* [male, 48 years, 1.65]

**Theme 4: feedback helped motivate and find personalized solutions to increase physical activity**

- *So, I knew that I had to just get back into doing something...And having that monitor was almost like a critical friend, it was there to say ‘you can do this.’* [female, 48 years, 1.55]
- *Yes, that really helped and then over the six-week period, every week I was trying to do a little bit more and like I say, it’s not very difficult to do it’s just that now you are conscious of it and you are aware of it that you have to achieve so many steps per day.* [male, 48 years, 1.87]
- *Definitely. Anything is worth it. Any...Any activity, it doesn’t have to be gym five days a week. If I wasn’t doing five days a week at the gym, I didn’t consider myself to be active, basically. So now I know that because I wasn’t training five days a week, and I was actually able to show some green lights when I wasn’t doing the fi...it makes me realise that all of it counts. It’s completely changed. I’m actually more active because I’m down on myself for not doing five days a week at the gym.* [female, 44 years, 1.52]
- *And it’s achievable without knocking myself flat you know I can do it in little steps and I can move myself forward in little ways rather than try and charge at a wall and break through a wall. It is much easier that way. So again, using the word empowerment it has sort of empowered me into thinking I could do this.* [male, 54 years, 1.39]

**Theme 5: real-time feedback prompted attainment of acute daily goals**

- *Um, and I did find it motivating, and I did, um, you know, I was known to leave the house at kind of five minutes to bedtime to walk around the block at the time...Or spend five minutes doing star jumps to try and get vigorous activity in. So yes, having the targets I found very helpful, and yeah, and motivating and interesting and fun.* [female, 56 years, 1.56]
- *Um, I did actually, I surprised myself in how easy it was to make step goals. I did not think I walked that much but as soon as I was just tracking it, it was like “actually I’m not far off daily amounts if I just do a little bit more and better hit that target.* [male, 41 years, 1.53]

**Theme 6: now able to fit more physical activity into routine**

- *Making a conscious almost, not a plan, plan is probably a bit too grand, but saying ‘right each week I must do a certain amount of activity’ and I plan that and think about it and so...The type of person I am, I’m quite a sort of structured and quite organised person so just building that into my routine is a change in my behaviour.* [male, 53 years, 1.86]

**Theme 7: injury and illness hampered progress during intervention**

- *Right, um, it was slightly complicated by the fact that I was ill right in the middle of it so...I started off really motivated and felt really good about it and it was building very well. And then unfortunately, after about a month I guess, I got this fluey type thing, which really did kick in and, made it a bit of a struggle to do as much and build as much as I wanted to do it. And then of course it’s sort of came to the end of the programme really so I don’t feel like I did it as much justice as I would have liked to have done.* [female, 67 years, 1.58]

**Theme 8: felt confident in keeping up or increase physical activity further**

- *Yeah, it definitely made me think a bit more about the moderate bouts of activity and how important they are. And it made me more keen to do things like walking the dogs and, you know, walks to school and I wouldn’t the thought that to be useful before. And I think ‘oh they are quite a useful way of getting in extra steps.’* [female, 43 years, 1.71]

**Theme 9: intervention led to improved confidence and enjoyment of physical activity**

- *Yeah. I mean, variety...yeah, I think that’s been really helpful, actually. Because it’s less boring and, um, you don’t perceive it as...I think my perception of what exercise was and what it actually is very different now. So now activity isn’t exercise. Activity is just anything.* [female, 45 years, 1.52]
- *Knowing how more confident I am, which I, perhaps if I wasn’t recording it somewhere I wouldn’t have been aware of that...So that’s erm, yeah that’s a nice position to be in, having seen confidence increase with various things, various types of activities, it’s a nice position to be in for sort of in the future.* [female, 48 years, 1.51]

**Theme 10: intervention prompted participants to purchase or consider purchasing a real-time feedback device**

- *It has spurred me on to get one of these, to actually buy one of these Fitbits. Which is just going to continue to let me know in real-time exactly what I’m doing and very similar in fact it is in steps and calories burnt off and what have you and the fact everyone else in the office have got one.* [female, 60 years, 1.52]
- *Which I suppose sounds really obvious when you say it, but it hadn’t ever linked with me before. And, I now have a little Fitbit because I want to now...I’ve become slightly obsessed with steps.* [female, 63 years, 1.37]

**Theme 11: real-time feedback element considered the most useful element of the intervention**

- *The instant display I think is what...I mean, I did go online that that’s in retrospect, you didn't get to see that until you had already done it. Whereas in today’s society we want instant answers so having the display and being able to look at it, um, was, you know, was motivating.* [female, 62 years, 1.25]
- *Um, the, the monitoring device, I found I used the little tiny daily, daily monitor, all the time...that was almost obsessive!* [male, 46 years, 1.48]

**Theme 12: web-based feedback useful initial picture**

- *You see on the computer screen and it was just flat line, I think that that is, visually, or when you look at it and you look at the figures and you look at that...that had probably quite an impact. And I think that that is...Probably the wake-up call which will remain with me, yeah, visually seeing it.* [male, 64 years, 1.72]

**Theme 13: web-based component could be improved**

- *Because I could only look at it at certain times at home without being able to do it when I wanted to do it was frustrating...If you see what I mean? So, just only having a sort of biweekly uploaded my information...I wish I could have just done it as and when and seen more feedback.* [male, 41 years, 1.53]
- *I think to be perfectly honest it was...it was sort of time element of it. I didn’t feel that I had the time just to sit and...and look at it, which I probably should have done, but it felt as if the more instantaneous response from the monitor was actually...or the display was...was what I needed on a day-to-day basis.* [female, 52 years, 1.64]

**Theme 14: some issues with device comfort or data trust**

- *The exercise I tend to do is like cycling and the bottom half of my body it probably won’t show a great deal of vigorous activity. Which, okay it is the limitation of the technology and the technology at that time, but I was mildly irritated by that.* [male, 55 years, 1.50]

**Theme 15: suggested improvements**

- *10,000 steps is nice and easy cause that’s just walking, you can just incorporate that into your daily activity, but then the vigorous activity, I could do it if I go for a run, but any other way I wouldn’t know. I only had ideas of cycling and rowing and though there are suggestions, but a programme of how you can achieve them would have been helpful.* [female, 45 years, 1.60]
- *So, if you said to me your cholesterol is 5 at the end of the study you told me my cholesterol...well...I found out my cholesterol...because you know cholesterol response to exercise had dropped to 3.5 that would have been a big encouragement.* [female, 55 years, 1.61]

Most of the intervention group felt that they were now being more proactive about fitting PA into their routine after the intervention (theme 6), whereas a handful of participants alleged to have had an illness or injury during the program that hampered their progress (theme 7). Approximately two-thirds of participants expressed further intentions to take up new, or perform more, PA, and approximately half of the group felt confident that they could maintain their PA levels after the program and had made lasting behavioral changes (theme 8). In addition, some participants felt that they had improved their confidence and sense of competence for PA, whereas others expressed a greater enjoyment for PA and an improved sense of health and well-being (theme 9). Many participants said that they missed not having the real-time activity monitor once it was removed after 6 weeks, and by the time of the interview, approximately one-third of participants had purchased a commercial PA tracker for personal use, with many more considering acquiring a device (theme 10).

For many of the intervention participants, the real-time display was a favorable component for the self-monitoring of activity and more important than website feedback (theme 11). That said, there was still a reasonable proportion of participants who made reference to the multidimensional feedback as a useful way of viewing the overall picture, and some even described it as a wake-up call (theme 12). Some participants suggested that their engagement with the feedback on the web platform may have been improved if it was more readily available and that sitting down at a computer felt counterintuitive to being (more) physically active (theme 13). The data also revealed that for certain participants, there were minor issues with the device itself in terms of either trusting the feedback or its wearability (theme 14). Finally, a handful of participants made recommendations for the improved utility of the monitor and feedback system, which included the need for more prompts and guidance or links to their health data collected during assessment sessions to help them evaluate the impact of more PA or for motivation (theme 15).

## Discussion

### Principal Findings

In this exploratory RCT, we evaluated a 6-week intervention using personalized real-time digital PA feedback and sophisticated web-based multidimensional PA feedback combined with brief trainer support. Exploratory analysis demonstrated no change in PA between groups immediately after the intervention; however, improvements were found for several PA metrics that formed part of the feedback at the 12 weeks follow-up. Subgroup analysis suggests that this effect was more pronounced in female participants and in those with lower baseline activity levels. Very little changed in respect to secondary health outcomes, with the exception of insulin resistance and self-reported mental health, which showed signs of improvement after 12 weeks. Qualitative data suggest that participants found the multicomponent intervention informative and motivating, with the real-time feedback being heralded as the single most memorable and supportive component within the context of the overall treatment package.

### Comparison With Other Literature

A novel aspect of this study was the multidimensional approach that, we hypothesized, helps individuals to understand their behavior and find bespoke behavioral solutions for increasing their PA [16]. We hypothesized that using a multidimensional approach to PA promotion and feedback would provide options and self-endorsed choices to foster autonomous motivation and would satisfy the needs for autonomy and competence. Following the 6-week active phase of the intervention, we witnessed favorable improvements in autonomous versus controlled motivation, perceived competence, and barrier self-efficacy, which offers support for the proposed mechanism and the multidimensional approach. Moreover, the qualitative evaluation aligns with our previous development work, which found that receiving detailed, visual multidimensional PA feedback is helpful for raising awareness, understanding, and intention to change [17,20]. We hypothesized that the addition of real-time feedback might help translate those intentions into behavior [57,58]. However, beneficial differences in PA were only observed after a 6-week period in which the whole treatment package (including the real-time display) was removed rather than immediately after the active intervention. Participants expressed that they valued the real-time feedback during the interview more than other components of the intervention and highlighted that it empowered them to adjust their behavior on a more discrete basis as they strive toward a desired daily target (eg, serving as a prompt to take an additional 1000-step walk if they were short toward the end of a day).

Other studies have observed real-time feedback to be an effective tool for increasing PA when used in conjunction with detailed web-based feedback and trainer support. Vandelenotte et al [27] demonstrated that adding a Fitbit device to their theory-informed web-based PA intervention increased total PA and MVPA by up to 3 times relative to a nontracker, web-only group. Their study, whose website went beyond the provision of feedback to provide individually tailored advice on a number of self-regulation strategies, found that real-time monitoring also improved engagement and adherence to the main web content and the overall package of behavioral support. Similarly, a large RCT by Harris et al [25] found that combining brief nurse support, retrospective accelerometer feedback, and continuous pedometer feedback led to significant, sustained changes in PA in the intervention versus control groups at 3 and 12 months. In another trial, the same research team demonstrated that continuous pedometer feedback provided effective support both with and without trainer input versus a control group with no feedback or trainer support [24]. The effects observed in these studies, albeit modest in size, were maintained after 3- to 4-year follow-up periods [59], suggesting that technology-based PA interventions such as the one used in this study can help individuals make long-lasting changes.

Our qualitative findings corroborated key findings from the Pedometer And Consultation Evaluation-U and Pedometer Accelerometer Consultation Evaluation-Lift studies, which were conducted by Harris et al [24,25]. Specifically, participants who received the nurse-led pedometer intervention experienced greater awareness of the PA guidelines and their own PA levels. They also placed more importance on being active and helped participants to embed PA in their own routines [25,60,61]. Participants also found real-time feedback useful for initiating and monitoring behavior change in relation to personalized goals, and, mirroring the findings reported in the present work, some went on to invest in other wearable trackers after their intervention, although distrust in the accuracy was identified as a potential barrier to effectiveness [60]. A set of themes derived from this study (eg, illness and injury) and the work of Harris et al [25] (eg, weather and lack of time) was the fact that common external barriers still existed for participants that could not be overcome by the real-time feedback interventions. Recommendations from participants in these and other qualitative studies suggest that more interpersonal prompts and guidance, resources for planning activities, meaningful challenges, and links to health data may be avenues to overcome barriers and enhance intrinsic motivation and behavioral maintenance in real-time feedback-based interventions [62-64].

The incongruent findings observed at 6 (immediately after the intervention, no difference) and 12 weeks (after a 6-week follow-up, moderate to large effects) warrant further consideration. The assessment used in this study and most RCTs with device-based PA outcomes relied on weekly snapshots of participants’ behavior. The small sample size and variability around the mean scores of the control group suggest that any fluctuations might be because of noise in the assessment. In the intervention group, a weekly snapshot may not give an accurate representation of a person’s true behavior [65]. Continuous measurement in both intervention and control groups would help decipher whether the 6-week observation is, for example, indicative of a dip in behavior following the removal of feedback, or whether the 12-week observations is, for example, indicative of the intervention participants learned rather than new habitual behavior. Given the advancing technology that enables long-term data capture, future studies would do well to investigate the stability and representativeness of PA behavior to guide trials on the most appropriate assessment window.

### Strengths and Limitations

The strengths of this study include the almost complete 24-7 objective, PA assessment and high compliance to the intervention, measured as the completeness of attendance to upload sessions and PA monitor wear time in the intervention group, and assessments in all groups (all 100%). The use of quantitative and qualitative evaluations also provides rich insights into the effectiveness of this approach. Limitations include the small sample size, short follow-up period, and use of a nonclinical population, which prevents the performance of more robust statistical analyses and means that any interpretation of these results should be viewed as preliminary rather than definitive and generalizable.

There is also a need to improve the synchronicity of the wearable devices as, in this study, technical issues meant that global web-based feedback could not be fully self-monitored without the trainer needing to recalibrate the personal targets and user profile used within the real-time display. This lack of autonomy over the web platform use may contribute to a more favorable evaluation of the real-time feedback element. Accordingly, we can determine neither the respective contributions of the real-time, web-based, or trainer support on individual participants’ behavior change nor whether favorable perceptions of the real-time element would have been the same without the more comprehensive web-based feedback. Recent meta-analyses of SDT-based techniques support the notion that different self-regulatory and trainer-delivered strategies may be useful for optimizing an individual’s motivation for PA [66,67]. Therefore, it is unlikely that any single component will be effective in isolation and that multicomponent interventions are required to provide optimal behavioral support. Nonetheless, future trials using more adaptable, multiple-group designs such as the multiphase optimization strategy would be advised to augment the complex intervention and evaluate the relative and complementary importance of the different elements [68].

### Conclusions

In conclusion, this exploratory RCT represents the first attempt at combining multidimensional feedback with real-time data and light touch trainer support across several important health-harnessing dimensions of PA as a means of helping individuals change their behavior. The results suggest that this approach may be a useful strategy for helping individuals with low levels of PA change their behavior. These findings can inform the design of future studies with larger and more diverse sample sizes, detailed process evaluations, and longer follow-up periods to explore the effectiveness of real-time, multidimensional feedback.

## Appendix

Multimedia Appendix 1 End-of-study semistructured interview topic guide and evaluation form.

Multimedia Appendix 2 Subgroup data for physical activity outcomes.

## Abbreviations

EQ: EuroQol
MET: metabolic equivalent of task
MIPACT: Multidimensional Individualized Physical Activity
MVPA: moderate to vigorous physical activity
PA: physical activity
PAL: physical activity level
RCT: randomized controlled trial
SDT: self-determination theory
SF-36: Short Form-36
