# Supplementary material for: Supporting Behavior Change in Sedentary Adults via Real-time Multidimensional Physical Activity Feedback: Mixed Methods Randomized Controlled Trial
Source: JMIR Form Res. 2022 Mar 2;6(3):e26525. doi: 10.2196/26525 (PMC8928046; doi:10.2196/26525)

**Multimedia Appendix 1. End of Study semi-structured interview topic guide and elevation form.**

**Topic guide**


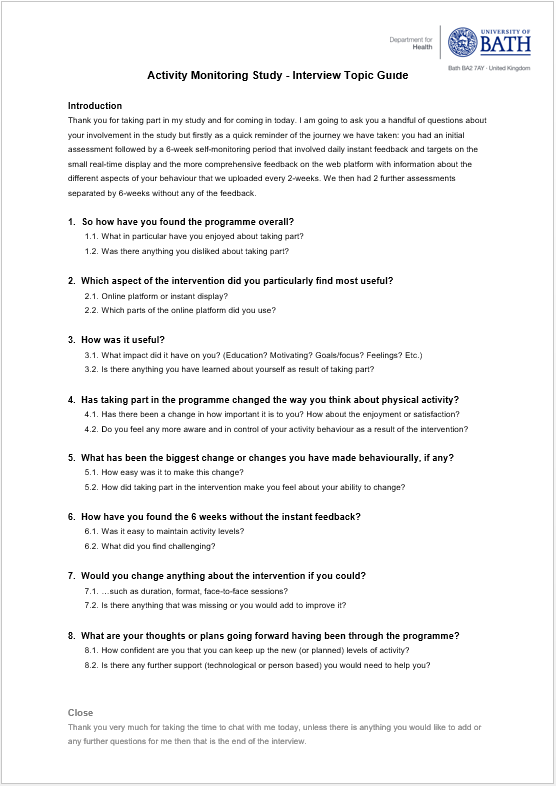


**End of intervention evaluation**


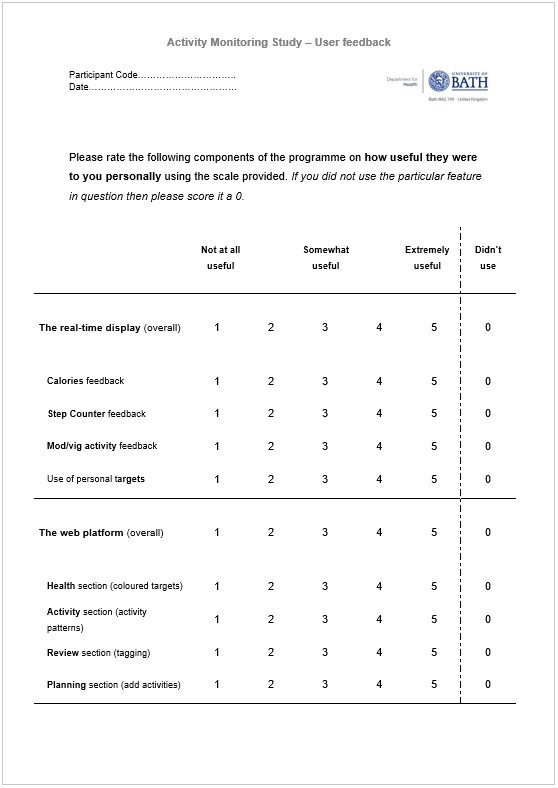

Supplement: Multimedia Appendix 1 [file formative_v6i3e26525_app1.docx]
