# Supplementary material for: Supporting Behavior Change in Sedentary Adults via Real-time Multidimensional Physical Activity Feedback: Mixed Methods Randomized Controlled Trial
Source: JMIR Form Res. 2022 Mar 2;6(3):e26525. doi: 10.2196/26525 (PMC8928046; doi:10.2196/26525)
Supplement: Multimedia Appendix 2 [file formative_v6i3e26525_app2.docx]

**Table S****1. Subgroup analysis sex and baseline PAL using a cut point of 1.6. The scores represent the raw unadjusted mean (SD) 12-week change in each physical activity outcome.**

| **Outcome** |  | **Sex** | | **Baseline PAL** | |
| --- | --- | --- | --- | --- | --- |
|  |  | **Female** | **Male** | **Inactive (<1.6)** | **Active (≥1.6)** |
| Sedentary | INT | -55 (67) | -2 (62) | -55 (67) | -4 (63) |
| (min/day) | CON | 13 (44) | 7 (61) | 9 (45) | 11 (58) |
| Moderate | INT | 29 (44) | 6 (42) | 31 (39) | 5 (45) |
| (min/day) | CON | -14 (19) | 3 (41) | -10 (17) | -4 (41) |
| PAL | INT | 0.09 (0.11) | 0.03 (0.14) | 0.11 (0.10) | 0.01 (0.14) |
| (TEE/BMR) | CON | -0.03 (0.08) | -0.02 (0.12) | -0.02 (0.07) | -0.03 (0.12) |
| MVPA bouts | INT | 133 (275) | 63 (271) | 180 (222) | 15 (301) |
| (min/week) | CON | -115 (70) | -9 (229) | -90 (90) | -44 (218) |
| Vigorous | INT | 6 (32) | 26 (58) | 12 (22) | 18 (64) |
| (min/week) | CON | -5 (27) | -5 (44) | -5 (29) | -5 (41) |
| Steps | INT | 1568 (2424) | 648 (1993) | 1593 (2478) | 674 (1947) |
| (steps/day) | CON | -253 (1238) | -442 (1047) | -174 (1179) | -488 (1116) |

PAL = physical activity level; TEE = total energy expenditure; BMR = basal metabolic rate;

MVPA = moderate to vigorous intensity physical activity; INT = intervention group; CON = control group

**Table S2 - Mean (SD) age, body mass, and physical activity level of male and female participants in the intervention and control groups.**

|  |  | **Females** | **Males** | **Inactive**  **(PAL <1.6)** | **Active**  **(PAL ≥1.6)** |
| --- | --- | --- | --- | --- | --- |
| **N** | Total | 28 | 23 | 26 | 25 |
|  | INT | 20 | 16 | 20 | 16 |
|  | CON | 8 | 7 | 7 | 8 |
| **Age**  **(years)** | Total | 51.1 (7.5) | 54.3 (8.9) | 52.0 (8.2) | 53.2 (8.4) |
|  | INT | 51.7 (7.9) | 55.0 (8.4) | 53.4 (8.3) | 52.4 (8.3) |
|  | CON | 49.5 (6.5) | 52.9 (10.5) | 48.0 (6.8) | 53.8 (9.3) |
| **Baseline BMI**  **(kg/m^2^)** | Total | 27.7 (4.7) | 28.4 (5.1) | 28.4 (5.4) | 27.7 (4.2) |
|  | INT | 27.8 (4.8) | 29.1 (4.9) | 29.0 (5.3) | 27.6 (4.3) |
|  | CON | 27.6 (4.7) | 26.9 (5.5) | 26.6 (5.7) | 27.8 (4.5) |
| **Baseline PAL**  **(TEE/BMR)** | Total | 1.56 (0.14) | 1.66 (0.14) | 1.50 (0.08) | 1.74 (0.09) |
|  | INT | 1.57 (0.16) | 1.65 (0.16) | 1.49 (0.09) | 1.74 (0.10) |
|  | CON | 1.55 (0.12) | 1.70 (0.08) | 1.51 (0.08) | 1.72 (0.06) |

PAL = physical activity level; TEE = total energy expenditure; BMR = basal metabolic rate;

BMI = body mass index; INT = intervention group; CON = control group
